# Meal-specific dietary patterns relate to memory functioning

**DOI:** 10.3389/fnut.2026.1760033

**Published:** 2026-04-28

**Authors:** Maria Kossowska-Wywiał, Oksana Kublanova, Adrianna Zając, Jakub Orłowski, Greta Front, Piotr Kulisz, Paweł Maciejewski, Weronika Bylina, Aneta Brzezicka

**Affiliations:** Institute of Psychology, SWPS University, Warsaw, Poland

**Keywords:** cognitive function, episodic recognition memory, evening eating patterns, meal-specific dietary analysis, western-style diet

## Abstract

This research addresses a critical gap in our understanding of how specific meals consumed during the day relate to cognitive function, particularly memory performance. While numerous studies have explored overall dietary patterns, few have considered the isolated effects of specific meals. Across two studies, we investigated subjective and objective measures of memory in relation to dietary habits and meal-specific consumption among adults aged 20 to 90 years. In Study 1, individuals consuming higher amounts of foods with potential adverse health effects, especially during supper, reported significantly poorer subjective memory functioning. Cheese consumption at supper emerged as the strongest predictor, with frequent cheese eaters rating their memory performance noticeably worse. Study 2 extended these findings by examining objective memory performance. Both Western-style and animal-based suppers were independently associated with poorer episodic recognition memory, with the strongest negative effects observed in younger adults. Detailed analysis revealed milk-based products and sweets as the most detrimental supper components for episodic recognition memory performance. Contrary to expectations, adherence to plant-based diet did not mitigate the negative effects of unhealthy supper choices on cognitive function. Our findings emphasize the importance of meal-specific dietary analysis. Recognizing meal-specific patterns may provide a more accurate understanding of how individual meals are associated with overall diet quality and cognitive functioning.

## Introduction

Understanding how food consumption affects cognitive function is an important area of research, yet most studies have focused on overall dietary patterns rather than the specific impact of individual meals consumed throughout the day.

Existing research on the relationship between meals and cognition has predominantly focused on breakfast, particularly its effects on cognitive performance in children, adolescents, and the elderly. These studies, which include both observational and randomized controlled trials, have examined how breakfast composition influences cognition (1); for adults: Galioto et al. (2); for children and adolescents: Adolphus et al. (3), as well as the effects of eating vs. skipping breakfast (2–4). In contrast, research on lunch and cognition is limited, with some studies addressing meal composition (5) and others comparing lunch consumption to omission (6). Only two studies have investigated nighttime eating in relation to cognition – one examining the composition of nighttime

food intake and its association with executive functioning (7), and another exploring the impact of nighttime energy intake on cognitive performance in older adults (8). Notably, no research to date has specifically isolated the cognitive effects of dinner or supper.

The relationship between overall dietary patterns and cognition has been extensively studied. Certain dietary patterns, such as the Mediterranean diet and the MIND diet, are recognized as supportive of brain health [for a review, see (9)], whereas others, such as the WD, have been linked to cognitive decline. There are no clear criteria for WD definition, yet it can be described as a diet high in saturated and trans fats (35–60% of total calories) and added sugar (10). Among brain regions, the hippocampus, a structure in the medial temporal lobe crucial for learning and memory, appears particularly vulnerable to the adverse effects of this diet. WD-induced inflammation and metabolic dysfunction disproportionately affect the hippocampus, leading to impairments in memory-related functions (11). Animal studies have well-documented that overconsumption of high-fat diet affects hippocampus-dependent cognitive processes (12). Although evidence from human studies remains limited, existing findings suggest that greater adherence to the WD is associated with poorer performance on memory tasks sensitive to hippocampal functioning (13–19). This association has been observed across all age groups, from prepubescent children (20) to the elderly (21). Additionally, in adults, an adherence to WD has also been linked to smaller hippocampal volume (22, 23).

The generalizability of research linking the WD to cognitive performance is limited. For example, a pioneering experimental study on the short-term effects of a WD in humans concluded that WD impairs hippocampal-dependent cognitive functioning, yet the study only manipulated a single meal – breakfast – rather than overall dietary patterns. In this study, participants who consumed a breakfast high in saturated fat and added sugar for four consecutive days showed significantly poorer recall of a 12-word list than a control group who consumed a breakfast lower in saturated fat and added sugar (17). This underscores the importance of considering cognitive effects in the context of specific meals, rather than attributing findings to broader dietary patterns. Overlooking meal-specific influences risks either overgeneralizing the effects of a single meal to an entire diet or underestimating its impact on cognition.

Building on evidence that Western dietary patterns may adversely affect functions sensitive to hippocampal integrity, the present study examined how both overall and meal-specific dietary patterns relate to memory performance. For the purposes of this study, meals were categorized as breakfast, dinner, and supper, in line with the conventional meal pattern in Poland, where breakfast refers to the first meal consumed in the morning, dinner to the afternoon meal, and supper to the final meal consumed in the evening. Memory functioning was assessed using both objective and subjective measures, including episodic recognition memory, which is typically supported by medial temporal lobe systems, and short-term memory tasks, which are relatively less dependent on hippocampal functioning. Given growing evidence that diet may either mitigate (24) or accelerate age-related cognitive decline (9), and considering the well-established association between aging and cognitive decline, age was included as a covariate in all analyses. Specifically, this study addressed three main research questions: (1) Are overall dietary patterns and specific meals associated with the quality of memory functioning? (2) Do these associations differ across age groups? (3) Which specific dietary components are most strongly linked to memory performance?

## Materials and methods

### Ethics

Both studies received ethical approval (No. 30/2023) from the Ethics Committee of SWPS University. All participants provided informed consent electronically prior to participation, and the consent procedure adhered to the principles of the Declaration of Helsinki. The participation was voluntary, with participants free to withdraw at any time.

### Study 1. Experimental design

Study 1 was a preliminary, hypothesis-generating online survey to examine associations between daily dietary habits, meal characteristics, and self-perceived memory functioning in everyday life. Data was collected using the Qualtrics platform (25). A total of 77 individuals participated (Table 1), including 39 women, 37 men, and one person who chose not to disclose their gender. The age of respondents ranged from 20 to 60 years. No exclusion criteria were applied. The recruitment process was conducted online through Facebook groups between June 2023 and June 2025.

**Table 1:** Summary of participant characteristics and descriptive statistics in study 1.

Characteristics	Number (%) (*N* = 77)
Sex	
Women	39 (50.6%)
Men	37 (48.1%)
Unknown	1 (1.3%)
Age	
Minimum	20
Maximum	60
Mean	37
SD	10.5
Education	
Vocational	5 (6.5%)
Secondary	31 (40.3%)
Higher	41 (53.2%)

SD, standard deviation; BMI, Body Mass Index.

### Daily dietary habits and individual meals patterns

To investigate dietary behaviors, we employed the Polish Questionnaire of Eating and Behaviors (QEB) (26). We focused on the section assessing foods consumed for breakfast, dinner, and supper when these food items or meals were eaten at least three times per week, to capture regular or habitual consumption (27, 28). To quantify non-healthy-diet-index, we developed composite indicators reflecting both overall dietary habits and meal-specific consumption patterns. These indicators were based on the frequency of consuming predefined food items classified as “healthy” or “non-healthy” according to the literature-based Polish FFQ and KomPAN frameworks developed by Wadołowska et al. (27) and Jeżewska-Zychowicz et al. (28), respectively. This classification was established for food products and dietary habits typical of the Polish population. In this context, “healthy” and “non-healthy” refer to food items classified within the questionnaire as representing dietary patterns with potentially more or less favorable health effects when consumed frequently, rather than implying that these foods are inherently healthy or unhealthy in every dietary context. Meal-specific dietary indices were created separately. For breakfast, the non-healthy-breakfast-index included meat, processed meat, cold cuts, cheese (solid yellow or spreadable), bread, cereal products, jam or honey, and juice. For dinner, the non-healthy-dinner-index included meat, processed meat, cold cuts, flour-based products (e.g., pancakes or dumplings), bread, cereal products, desserts, and juice. For supper, the non-healthy-supper-index included meat, processed meat, cold cuts, cheese, bread, jam or honey, and juice. The non-healthy-diet-index was calculated by summing foods with potentially non-healthy effects participants eat across breakfast, dinner, and supper.

### Subjective memory functioning questionnaire

Self-reported memory functioning was assessed using a self-translated version of the Memory Functioning Questionnaire [MFQ; (29)]. This measure was included to capture participants' evaluations of their everyday memory functioning, rather than objective long-term memory performance. It consists of four memory measures, on a 7-point Likert scale, reflecting the subjective assessment of the participant on the following dimensions: (1) retrospective functioning scale “How is your memory compared to the way it was a year ago/five years ago? etc., (2) frequency of forgetting: “How Often do these present a problem for you - names/faces? etc., (3) seriousness scale “When you actually forget in these situations, how serious of a problem do you consider the memory failures to be names/faces, etc.?,” (4) mnemonic usage scale “How often do you use these techniques to remind yourself about things? e.g., keep an appointment book/write yourself reminder notes, etc.” Finally, we created an overall index of the subjective memory functioning taking all four measures together. Each measure, as well as the overall index, was calculated by summing the scores of the individual items.

### Study 2. Experimental design

**Figure 1:**
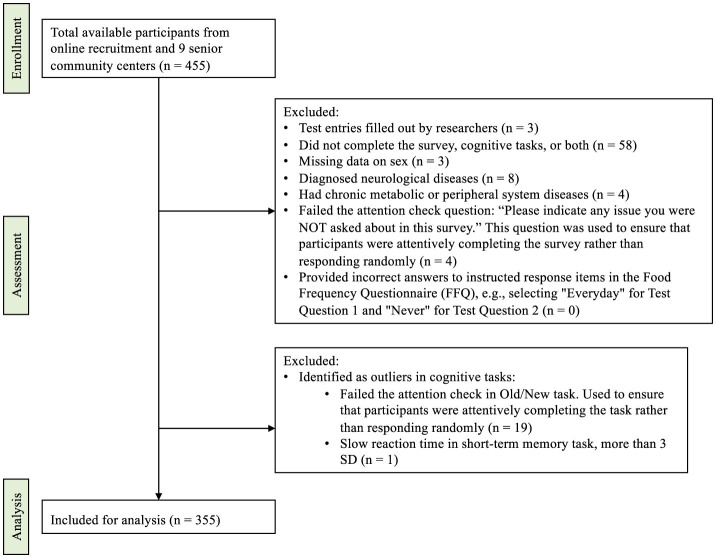
STROBE diagram of participants' flow.

In our second study, we conducted an online cross-sectional survey followed by two neuropsychological tasks to examine the relationship between objective memory functioning measures and daily dietary habits, as well as the characteristics of individual meals. Survey data was collected using the Qualtrics platform, the cognitive tasks were constructed in PsychoPy (30) and distributed via Pavlovia platform. A total of 355 individuals participated from Warsaw and surrounding areas, including 311 women, 44 men (Figure 1; Table 2). The age of respondents ranged from 20 to 90 years old. The participation was voluntary, with participants free to withdraw at any time between June 2023 and June 2025, recruitment was conducted both online and offline. Online, study information was shared via our lab's Facebook group and Warsaw district Facebook groups to recruit individuals living near the research site, as participants were also invited to a subsequent on-site RCT in Warsaw. To reach individuals who may not use the internet, printed posters and leaflets were also distributed in local community settings, such as libraries, cinemas, primary care clinics, and senior clubs.

**Table 2:** Summary of participant characteristics and descriptive statistics in study 2.

Characteristics	Number (%) (*N* = 355)
Sex	
Women	311 (87.6%)
Men	44 (12.4%)
Age	
Minimum	20
Maximum	90
Mean	40.25
SD	15.77
Employment	
Employed	250 (70.4%)
Unemployed	105 (29.6%)
Student	56 (15.8%)
Retirement	30 (8.5%)
Different	19 (5.4%)
Education	
Primary	0 (0%)
Secondary	82 (23.1%)
Higher	273 (76.9%)
BMI	
Minimum	13.84
Maximum	44.08
Mean	23.95
SD	4.35
Smoking	
Yes	36 (10.1%)
No	319 (89.9%)
Physical activity	
Sedentary or light	167 (47.0%)
Medium active	165 (46.5%)
Vigorously active	23 (6.5%)
Diet type	
Omnivores	279 (78.6%)
Vegetarians	59 (16.6%)
Vegans	17 (4.8%)
Antibiotic use	
Once a month	2 (0.6%)
Once every few months	9 (2.5%)
Once every six months	13 (3.7%)
Once a year	43 (12.1%)
Once every few years	263 (74.1%)
Never (so far)	25 (7.0%)
Type of birth	
Natural birth	293 (82.5%)
Cesarean section	53 (14.9%)
Unknown	9 (2.5%)

SD, standard deviation; BMI, Body Mass Index.

Participants who met specific exclusion criteria were removed from the analysis. A total of 58 individuals were excluded because they did not complete the survey, the cognitive tasks, or both. Eight participants were excluded due to diagnosed neurological diseases, including spinal muscular atrophy, subarachnoid cyst, multiple sclerosis, epilepsy, brain tumor, polyneuropathy, stroke, Parkinson's disease, or gastrointestinal syndrome. Four individuals were excluded due to chronic metabolic or peripheral system diseases, including SIBO and insulin resistance. Four participants were removed for providing incorrect answers to a control question that asked them to indicate the issue they had not yet been asked about in the survey, with the correct answer being “number of teeth.” No participants were excluded based on the second or third control question, which required marking specific responses in the food frequency questionnaire. Nineteen individuals were excluded for scoring below the threshold in the learning phase of the Old/New Recognition task, where a minimum of seven points out of eight was required, consistent with standard quality control procedures used to ensure adequate task engagement. One participant was removed due to an extremely slow reaction time in the short-term memory task, defined as exceeding three standard deviations above the mean. This final sample consisted of participants who met all inclusion criteria and provided complete and reliable data for further analysis.

### Western-style, animal-based and plant-based diet

**Figure 2:**
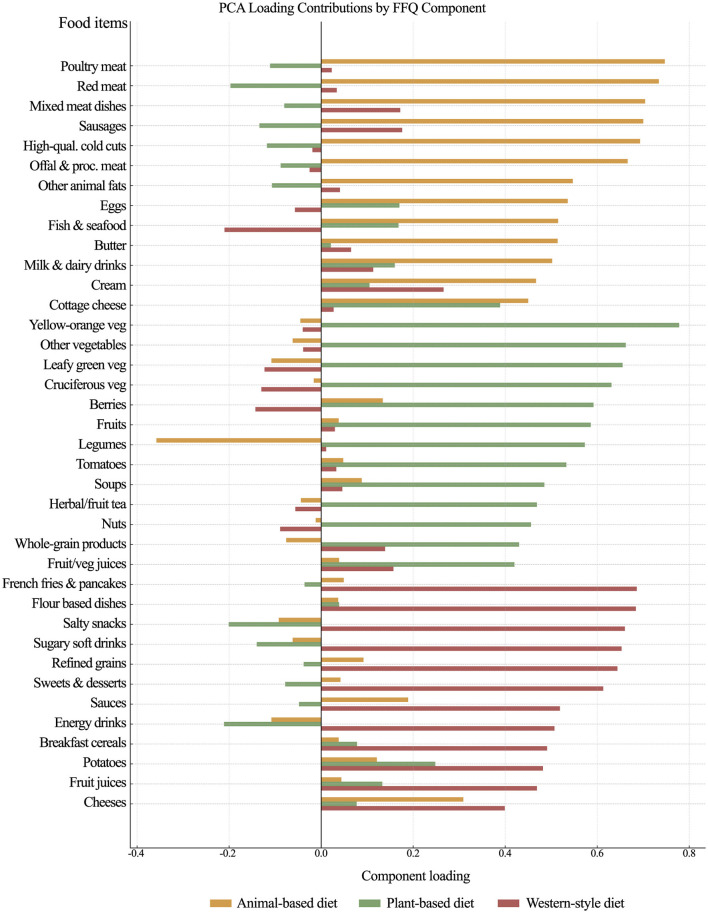
Dietary patterns extracted from principal component analysis. The figure shows the food items and their factor loadings for each dietary component from principal component analysis.

To assess dietary patterns, we administered the Food Frequency Questionnaire (FFQ) (31). The FFQ captured habitual dietary intake over the preceding year and included items covering 45 food products. For each product, respondents indicated their usual consumption frequency by selecting one of six categories: “never,” “1–3 times per month,” “once a week,” “several times a week,” “once a day,” or “several times a day.” A principal component analysis (PCA) conducted on the frequency of consumption of 45 group products extracted three components, explaining 37.40% of the total variance. The scree plot suggested retaining three components, as there was a noticeable inflection point after the third component, indicating a plateau in the eigenvalues. The first component explained 14.99% of the variance, the second explained 12.29%, and the third explained 10.13%. The first component, labeled animal-based products, reflected a diet high in various meats and animal products. The second component, plant-based products, reflected a diet rich in different types of vegetables, fruit and plant-based products. The third component, western-style diet highlighted consumption of processed foods. The rotated component matrix of the food items is presented in Supplementary Table S1 (in the Supplementary Materials) and illustrated in Figure 2.

### Control check: identified diet components correlate with dietary quality index

We conducted a control check to assess how the identified dietary components correlate with a literature-derived dietary quality index (DQI) based on the KomPAN questionnaire (28). The detailed mathematical procedure for calculating the DQI is provided in the original reference and is not presented here, as the index was used solely as a control check. The analysis confirmed that the three dietary components align with established dietary quality indices. A plant-based diet showed a strong positive correlation with DQI [r_s_(353) = 0.823, *p* < 0.001, 95% CI (0.783, 0.857)], indicating that a higher plant-based diet score was associated with better diet quality. The analysis also revealed a moderate negative correlation between the Western-style diet and DQI [r_s_(353) = −0.345, *p* < 0.001, 95% CI (−0.434, −0.251)], suggesting that a higher Western-style diet score was linked to lower diet quality. Additionally, PCA is a well-established method for analyzing FFQ data (32).

### Western-style and animal-based supper

To create variables for animal-based and Western-style suppers, we applied a custom calculation method. These indicators were based on the frequency of consuming specific food items, usually for supper. The animal-based supper variable was generated by counting the consumption of milk-based products, dairy, eggs, fish, and meat. Similarly, the Western-style supper variable was created by counting the consumption of sweets, salty snacks, and alcohol.

### Episodic recognition memory task

**Figure 3:**
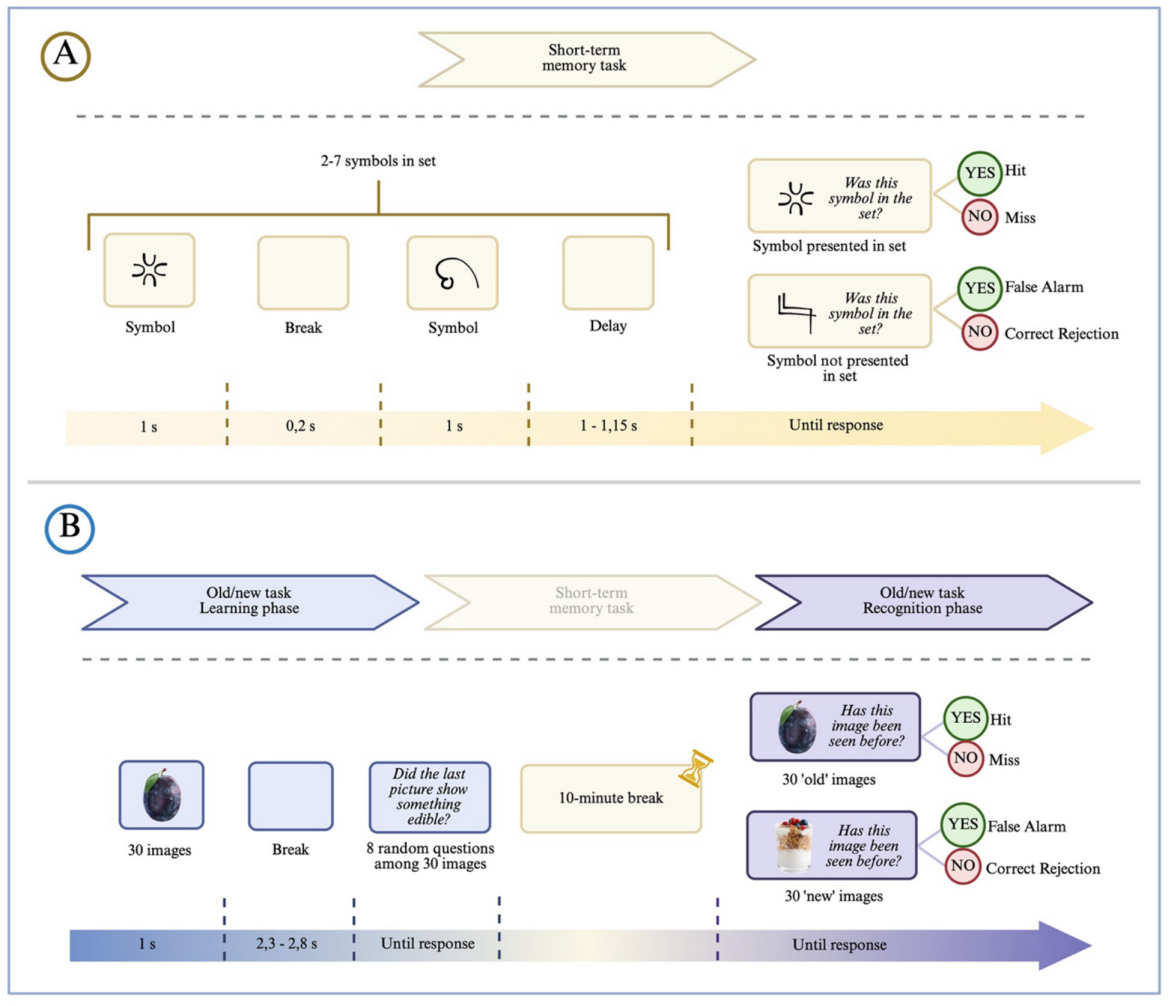
Schematic illustrating the short-term memory **(A)** and episodic recognition memory **(B)** task. **(A)** Subjects were shown a series of 2 to 7 abstract symbols, followed by a brief pause and a single probe symbol. They judged whether the probe had appeared in the preceding series. **(B)** Subjects were first shown 30 images during a learning phase, followed by a 10-minute break. In the recognition phase, they judged whether each of 60 images (30 old, 30 new) had been seen before. Created with BioRender.com.

We employed an “Old/New” Recognition task to examine episodic recognition memory (Figure 3), which consisted of two phases: a learning phase and a recognition phase. During the learning phase, participants viewed 30 pictures depicting healthy foods, healthy dishes, unhealthy foods, unhealthy dishes, and clothing items. On eight occasions, they were asked, “Did the last picture show something edible?” to maintain their attention. Following the learning phase, participants took a 10-min break during which they completed a short-term memory task described in the section below. In the recognition phase, participants were presented with 30 “old” and 30 “new” pictures in random order and were asked to determine whether each picture had been shown during the learning phase.

To analyse episodic recognition memory task we followed Macmillan and Creelman (33) signal detection theory, where d' is the ability to discriminate between signal and noise (between old and new stimuli), H stands for the proportion of correct changes detected (hit rate), and F stands for the proportion of changes incorrectly reported (false alarm rate).


$$\begin{array}{c} d^{\prime }=zH-zF \end{array}$$

### Short-term memory task

**Figure 4:**
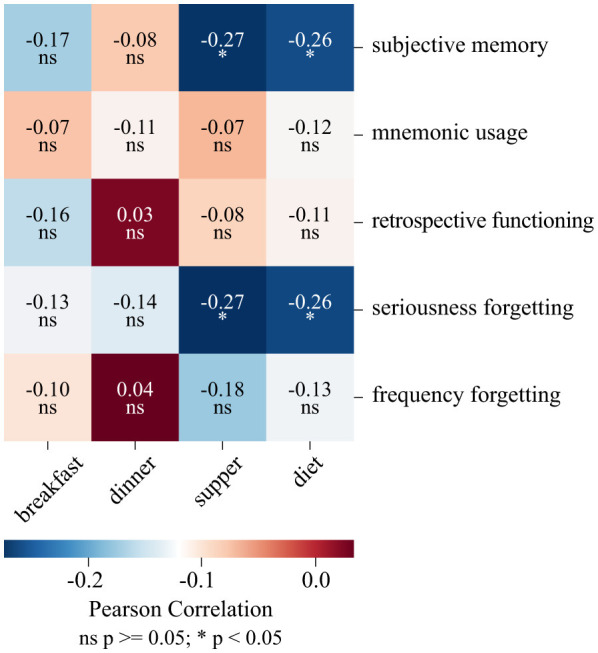
Correlations between dietary habits and subjective memory functioning Pearson correlation coefficients (r) between dietary habits categorized by non-healthy meal type (breakfast, dinner, supper) and the overall non-healthy diet index (diet) and key aspects of subjective memory functioning. Correlation strength is color-coded, with blue indicating negative and red indicating positive correlations. Significant relationships (*p* < 0.05) are marked with *, and non-significant ones (*p* ≥ 0.05) are labeled “ns.” Variables assessed include subjective memory, mnemonic usage, retrospective functioning, seriousness of forgetting, and frequency of forgetting.

We employed a modified Sternberg Task to assess short-term memory (Figure 4). In this task, participants were presented with a set of abstract symbols containing between 2 and 7 elements. Each series was followed by a brief pause, after which a single symbol appeared. Participants then had to decide whether the symbol had been part of the previously displayed series.

The data analysis was based on Vogel et al. (34), where K is the WM capacity, S represents the target array size (from 2 to 7), H represents the proportion of correct changes detected (hit rate), and F represents the proportion of changes incorrectly reported (false alarm rate).


$$\begin{array}{c} K=S^{*}\left(H-F\right) \end{array}$$

Based on K for each array size, we employed a custom calculation technique to calculate overall working memory load. It was determined by identifying the largest set size for which they consistently responded correctly.

### Statistical analysis

We examined the relationship between variables using either Pearson's or Spearman's rank-order correlation, depending on whether the data met the necessary assumptions. Scatterplots were visually inspected to determine if the relationship was linear (suitable for Pearson's correlation) or monotonic (suitable for Spearman's correlation). Normality was assessed using the Shapiro–Wilk test (with *p* > 0.05 indicating normality), and potential outliers were identified and examined.

We examined all food declarative intake responses using multiple linear regression models, with d′ serving as a continuous outcome variables and k load as an ordinal outcome variable. To assess linearity, we employed partial regression plots and plots of studentized residuals vs. predicted values. The independence of residuals was evaluated using the Durbin–Watson statistic, with values near 2 indicating no autocorrelation. Homoscedasticity was assessed through visual inspection of plots of studentized residuals against unstandardized predicted values. Multicollinearity was evaluated by ensuring that tolerance values exceeded 0.1. Standardized residuals were examined for values exceeding ±3 standard deviations; as the exclusion of these potential outliers did not change the results, they were retained in the analyses. We also inspected leverage values (flagging those above 0.2) and Cook's distance (flagging values above 1) to identify influential observations. Finally, normal Q–Q plots were used to verify the normality of the residuals.

We used moderation analysis to examine whether age modulates the association between dietary patterns and memory performance. Continuous variables defining interaction terms were mean-centered to reduce multicollinearity. To interpret moderation effects, we used percentile-based conditioning values at the 25th, 50th, and 75th percentiles of age. Additionally, the Johnson-Neyman (JN) technique was applied to identify the specific range of age values where the interaction effect was statistically significant.

We conducted a principal component analysis (PCA) to reduce data dimensionality. Sampling adequacy was confirmed using the Kaiser-Meyer-Olkin (KMO) statistic, which was 0.803, exceeding the recommended threshold of 0.6. Bartlett's Test of Sphericity was significant, χ^2^(1081) = 5844.08, *p* < 0.001, indicating that correlations among the variables were sufficient for PCA. We initially performed a Direct Oblimin rotation and examined the Component Correlation Matrix. As the intercomponent correlations were all below 0.32, an orthogonal rotation (Varimax) was deemed more appropriate (35). Statistical analysis were performed using IBM SPSS Statistics (36).

## Results

### Study 1. Subjective memory functioning in relation to overall dietary habits and specific meal types

#### Non-healthy diet index correlates with subjective memory functioning

We first examined the relationship between daily dietary habits and self-assessed memory. Specifically, we focused on the consumption of foods with potentially unhealthy effects and their association with participants' subjective memory evaluations. We found that participants who consumed more foods classified as non-healthy during the day reported poorer subjective memory functioning [r_(75)_ = −0.257, *p* = 0.024, 95% CI (−0.442, −0.029)]. This association was primarily reflected in how strongly they perceived their own forgetfulness [r_(75)_ = −0.26, *p* = 0.023, 95% CI (−0.434, −0.065)].

#### Non-healthy supper index is a stronger predictor of subjective memory functioning than non-healthy diet index

Next, we conducted a regression analysis to examine whether the consumption of foods classified as non-healthy at different meals predicts subjective memory functioning. We entered four predictors into the model: (1) non-healthy-breakfast-index, (2) non-healthy-dinner index, (3) non-healthy supper index, and (4) non-healthy-diet-index. Among these, only non-healthy supper index (*B* = −4.951; *p* = 0.017) significantly predicted subjective memory functioning, while the other variables show no meaningful effect. The final model accounted for 7.3% of the variation in subjective memory functioning. The analysis showed that people who consumed more foods with potential adverse health effects at supper tended to rate their memory as worse. Specifically, each additional food item classified as non-healthy and consumed at supper was associated with an approximately 4.95-point lower subjective memory score. Full regression coefficients and standard errors are presented in Table 3. To visualize this pattern, Figure 4. shows that higher non-healthy supper and overall diet indices were significantly associated with worse subjective memory and greater seriousness of forgetting (r ≈ −0.26, *p* < 0.05), while no comparable associations were observed for non-healthy breakfast or dinner. This consistency across methods highlights the robustness of the non-healthy supper effect.

**Table 3:** Summary of linear stepwise regression analysis for overall dietary habits and specific meal types predicting overall subjective memory index in the total sample (*N* = 77).

Stepwise regression on overall subjective memory index									
	B	*t*	*p*	95% CI for B		SE B	β	F statistics	R^2^
				LL	UL				
**Step 1**			0.02					5.922	0.073
Constant	171,126	41,072	< 0.001	162,826	179,426	4,167			
Non-healthy-supper-index	−4,951	−2,434	0.017	−9.003	−0.898	2.034	−0.271		
Excluded variables									
Non-healthy-dinner-index		−0.245	0.807				−0.103		
Non-healthy-diet-index		−0.778	0.439				−0.127		
Non-healthy-breakfast-index		−0.890	0.377				−0.028		

B, unstandardized regression coefficient; SE B, standard error of the unstandardized regression coefficient; β, standardized regression coefficient; ΔR2, change in the proportion of variance explained relative to Step 1. The dependent variable was the total score from the memory functioning questionnaire.

#### Cheese at supper is the strongest predictor of overall subjective memory functioning

Then, we wanted to disentangle the structure of the non-healthy supper index and assess the impact of its individual components. To do this, we tested five food classes: (1) meat (processed/cold cuts), (2) cheese (yellow or spreadable), (3) bread, (4) sweet preserves (jam/honey), and (5) juice. Among these, only cheese (*B* = −13,002; *p* = 0,011) was significantly associated with subjective memory functioning, while the other components did not show a meaningful effect. The final model accounted for 8.2% of the variation in subjective memory functioning. The prediction equation was: Overall subjective memory index score = 168,196–13,002 × cheese. The analysis revealed that participants who consumed cheese at supper at least three times per week tended to rate their memory as worse. Specifically, their self-reported memory scores were about 13 points lower compared to those who ate cheese less frequently. Full regression coefficients and standard errors are presented in Table 4.

**Table 4:** Summary of linear stepwise regression analysis for individual components of non-healthy supper index predicting overall subjective memory index in the total sample (*N* = 77).

Stepwise regression on overall subjective memory index									
	B	t	*p*	95% CI for B		SE B	β	F statistics	R^2^
				LL	UL				
**Step 1**			0.011					6.730	0.082
Constant	168.196	52.890	< 0.001	161.861	174.531	3.180			
Cheese	−13.002	−2.594	0.011	−22.986	−3.018	5.012	−0.287		
Excluded variables									
Sweet preserves (jam/honey)		−0.551	0.583				−0.630		
Meat (processed/cold cuts)		−0.697	0.488				−0.081		
Bread		−1.171	0.245				−0.133		
Juice		1.308	0.195				0.145		

B, unstandardized regression coefficient; SE B, standard error of the unstandardized regression coefficient; β, standardized regression coefficient; ΔR2, change in the proportion of variance explained relative to Step 1. The dependent variable was the total score from the memory functioning questionnaire.

### Study 2. Objective memory functioning in relation to daily dietary habits and evening food choices

#### Association between diet and memory

Study 2 was designed to build on the exploratory findings of Study 1, in which greater consumption of foods with potentially less favorable health effects was associated with poorer self-reported memory functioning, by testing whether a comparable relationship could also be detected using objective memory measures in an independent sample. This design was additionally informed by prior research showing that Western dietary patterns may impair hippocampal function and episodic recognition memory. At the same time, we broadened the scope of the analysis by examining three dietary patterns identified using the Food Frequency Questionnaire [KomPAN; (28)]: animal-based, plant-based, and Western-style diets, the latter corresponding conceptually to the non-healthy diet index from Study 1. We then assessed whether adherence to these patterns was associated with performance on short-term and episodic recognition memory tasks.

#### 3.2.2 . Western-style diet and objective of memory functioning

Firstly, we examined whether adherence to a Western-style dietary pattern is associated

with episodic recognition memory performance. While our initial findings indicated that individuals following this dietary pattern subjectively perceived their memory as weaker, this was not rue in the case of objective cognitive performance - those with an adherence to Western-style dietary pattern did not exhibit significantly lower scores on episodic recognition memory tasks (B = −0.054; *p* = 0.239). Furthermore, we assessed whether this relationship varied across age groups but found no evidence of age-related differences (interaction effect Western-style diet × age; B = 0.001; *p* = 0.682). These results suggest that, within this sample, an adherence to Western-style dietary pattern may not be linked to episodic recognition memory performance. Next, we explored whether an adherence to Western-style diet is linked to short-term memory performance. Our findings revealed that individuals who adhered to this diet had lower capacity of short-term memory (B = −0.258; *p* = 0.014). Interestingly, this contradicts our initial assumption that short-term memory - not being dependent on hippocampal function - would not be associated with Western diet. Additionally, age did not appear to moderate this relationship (interaction effect Western-style diet × age; B = −0.012; *p* = 0.086), as the association between an adherence to Western-style dietary patterns and short-term memory remained stable across different age groups. These findings indicate that, within this sample, adherence to a Western-style dietary patterns is associated with short-term memory performance, regardless of age. Full regression coefficients and standard errors are presented in Table 5.

**Table 5:** Summary of linear regression models predicting episodic recognition memory and short-term memory performance from age, adherence to a Western-style diet, and their interaction (Western-style diet × age) in the total sample (*N* = 355).

Outcome variable: episodic recognition memory score								
	B	SE	t	*p*	95% CI for B		F statistics	R^2^
					LL	UL		
**Model summary**				< 0.001			lip.51	0.097
Constant	2.968	0.335	8.859	< 0.001	2.309	3.627		
Predictors								
Western-style diet	−0.054	0.046	−1.18	0.239	−0.145	0.036		
Age	−0.012	0.003	−3.957	< 0.001	−0.018	−0.006		
Western-style diet × Age	0.001	0.003	0.41	0.682	−0.005	0.007		
Covariates								
Sex	−0.446	0.127	−3.525	< 0.001	−0.695	−0.197		
Education	0.217	0.102	2.128	0.034	0.017	0.418		
Outcome variable: short-term memory capacity								
	B	SE	t	*p*	95% CI for B		F statistics	R^2^
					LL	UL		
**Model summary**				< 0.001			9.419	0.119
Constant	4.899	0.757	6.469	< 0.001	3.409	6.389		
Predictors								
Western-style diet	−0.258	0.104	−2.473	0.014	−0.463	−0.053		
Age	−0.045	0.007	−6.69	< 0.001	−0.058	−0.032		
Western-style diet × Age	−0.012	0.007	−1.724	0.086	−0.025	0.002		
Covariates								
Sex	−0.101	0.286	−0.354	0.724	−0.664	0.461		
Education	0.123	0.231	0.531	0.595	−0.331	0.576		

B, unstandardized regression coefficient; SE B, standard error of the unstandardized regression coefficient; β, standardized regression coefficient. The dependent variable was the d' score from the episodic recognition memory task and the capacity score from short-term memory task.

#### Animal-based diet and measures of memory functioning

We first investigated whether an adherence to animal-based dietary pattern is linked to

episodic recognition memory performance. Our analysis suggests that this dietary pattern does not have a clear association with how well individuals perform on episodic recognition memory tasks (B = −0.034; *p* = 0.441). Additionally, age did not appear to moderate this relationship (interaction effect animal-based diet × age; B = 0.007; *p* = 0.805), meaning that the lack of association between an animal-based diet and episodic recognition memory remained consistent across different age groups. These findings indicate that, at least within this sample, consuming an animal-based diet does not seem to have an association with episodic recognition memory, regardless of age.

Next, we examined the relationship between an adherence to animal-based dietary patterns and short-term memory performance. No significant overall association was found between this dietary pattern and short-term memory capacity score (B = −0.044; *p* = 0.667). Additionally, age did not appear to moderate this relationship (interaction effect animal-based diet × age; B = −0.004; *p* = 0.593), as the association between an

adherence to animal-based dietary patterns and short-term memory remained stable across different age groups. These findings indicate that, within this sample, adherence to an animal-based diet is not associated with short-term memory performance, regardless of age. Full regression coefficients and standard errors are presented in Table 6.

**Table 6:** Summary of linear regression models predicting episodic recognition memory and short-term memory performance from age, adherence to animal-based diet, and their interaction (animal-based diet × age) in the total sample (*N* = 355).

Outcome variable: episodic recognition memory score								
	B	SE	t	*p*	95% CI for B		F statistics	R^2^
					LL	UL		
**Model summary**				< 0.001			7.315	0.095
Constant	2.852	0.328	8.703	< 0.001	2.208	3.497		
Predictors								
Animal-based diet	−0.034	0.044	−0.772	0.441	−0.121	0.053		
Age	−0.01	0.003	−3.558	< 0.001	−0.016	−0.005		
Animal-based diet × age	0.007	0.003	0.247	0.805	−0.005	0.006		
Covariates								
Sex	0.441	0.127	−3.487	< 0.001	−0.69	−0.192		
Education	0.253	0.101	2.505	0.013	0.054	0.451		
Outcome variable: short-term memory capacity								
	B	SE	t	*p*	95% CI for B		F statistics	R^2^
					LL	UL		
**Model summary**				< 0.001			7.606	0.098
Constant	5.278	0.749	7.maj	< 0.001	3.806	cze.75		
Predictors								
Animal-based diet	−0.044	0.101	−0.43	0.667	−0.243	0.156		
Age	−0.035	0.007	−5.352	< 0.001	−0.047	−0.022		
Animal-based × Age	−0.004	0.007	−0.534	0.593	−0.017	0.01		
Covariates								
Sex	−0.227	0.289	−0.785	0.433	−0.795	0.342		
Education	0.071	0.231	0.301	0.759	−0.383	0.524		

B, unstandardized regression coefficient; SE B, standard error of the unstandardized regression coefficient; β, standardized regression coefficient. The dependent variable was the d' score from the episodic recognition memory task and the capacity score from short-term memory task.

#### Plant-based diet and measures of memory functioning

We first examined whether an adherence to plant-based dietary pattern is linked to

episodic recognition memory performance. Our analysis indicated no clear association between this dietary pattern and performance on episodic recognition memory tasks (B = −0.001; *p* = 0.988). Additionally, age did not appear to moderate this relationship (interaction effect plant-based diet × age; B = −0.003; *p* = 0.334), meaning that the lack of association between an adherence to plant-based dietary pattern and episodic recognition memory remained consistent across different age groups. These findings suggest that, at least within this sample, following a plant-based dietary pattern does not seem to have an association with episodic recognition memory, regardless of age.

Next, we explored whether an adherence plant-based dietary pattern is linked to short-term memory performance. Our analysis did not reveal a significant association between this dietary pattern and short-term memory capacity score (B = −0.08; *p* = 0.491), suggesting that a plant-based diet alone may not have an association with short-term memory capacity. Additionally, age did not appear to moderate this relationship (interaction effect plant-based diet × age; B = −0.004; *p* = 0.484), as the lack of association between an adherence plant-based dietary pattern and short-term memory remained stable across different age groups. These findings indicate that, within this sample, adherence to a plant-based dietary pattern does not seem to be associated with short-term memory performance, regardless of age. Full regression coefficients and standard errors are presented in Table 7.

**Table 7:** Summary of linear regression models predicting episodic recognition memory and short-term memory performance from age, adherence to a plant-based diet, and their interaction (plant-based diet × age) in the total sample (*N* = 355).

Outcome variable: episodic recognition memory score								
	B	SE	t	*p*	95% CI for B		F statistics	R^2^
					LL	UL		
**Model summary**				< 0.001			7.359	0.095
Constant	2.923	0.319	9.171	< 0.001	2.297	3.550		
Predictors								
Plant-based diet	−0.001	0.043	−0.015	0.988	−0.086	0.085		
Age	−0.01	0.003	−3.665	< 0.001	−0.016	−0.005		
Plant-based diet × Age	−0.003	0.003	−0.967	0.334	−0.008	0.003		
Covariates								
Sex	−0.46	0.128	−3.611	< 0.001	−0.711	−0.21		
Education	0.24	0.099	2.429	0.016	0.046	0.434		
Outcome variable: short-term memory capacity								
	B	SE	t	*p*	95% CI for B		F statistics	R^2^
					LL	UL		
**Model summary**				< 0.001			7.748	0.1
Constant	5.295	0.728	7.277	< 0.001	3.864	6.727		
Predictors								
Plant-based diet	−0.08	0.099	−0.809	0.491	−0.274	0.115		
Age	−0.034	0.006	−5.421	< 0.001	−0.047	−0.022		
Plant-based diet × Age	−0.004	0.006	−0.7	0.484	−0.017	0.008		
Covariates								
Sex	−0.301	0.291	−1.034	0.302	−0.874	0.272		
Education	0.095	0.225	0.42	0.675	−0.349	0.537		

B, unstandardized regression coefficient; SE B, standard error of the unstandardized regression coefficient; β, standardized regression coefficient. The dependent variable was the d' score from the episodic recognition memory task and the capacity score from short-term memory task.

### Both animal-based supper and Western-style supper predict episodic recognition memory performance

Building on our earlier findings - where non-healthy-supper-index was identified as a key predictor of subjective memory functioning, with cheese emerging as the strongest factor - we sought to further examine how different food choices for supper contribute to objective memory performance. Specifically, we focused on two distinct supper patterns: Western-style suppers (including salty snacks, sweets, and alcohol) and animal-based suppers (consisting of meat, dairy, milk-based products, eggs, and fish), as both contribute to the non-healthy supper index. Our goal was to determine whether these dietary patterns are independently associated with episodic recognition memory performance or whether their effects interact. To investigate this, we conducted separate regression analyses for each supper type, tested for interaction effects, and controlled for age.

In the first step, we examined how Western-style supper relates to memory performance. While age alone accounted for a small portion of the variance in memory scores, adding Western-style supper significantly improved the model, revealing it as a significant negative predictor of episodic recognition memory (*B* = −0.219; *p* = 0.002). In the second step, a similar pattern was observed for animal-based supper, where including this dietary factor also strengthened the model, with animal-based supper emerging as a significant predictor (*B* = −0.104; *p* = 0.005).

In the third step, to gain a more comprehensive understanding, we tested a combined model that included both supper patterns and their interaction. The final model explained 9% of the variance in memory performance, with both Western-style supper (*B* = −0.209; *p* = 0.029) and animal-based supper (*B* = −0.104; *p* = 0.011) remaining significant predictors. Yet, their interaction was not significant, indicating that these dietary patterns do not amplify or modify each other's effects but rather contribute separately to episodic recognition memory score.

These findings highlight the complex ways in which diet may be associated with memory functioning. While both Western-style and animal-based suppers appear to have independent negative effects, they likely are associated with cognitive function through separate mechanisms rather than interacting with one another. Full regression coefficients and standard errors are presented in Table 8.

**Table 8:** Summary of linear regression models predicting episodic recognition memory performance from age, adherence to a western-style and animal-based supper and their interaction (Western-style supper × animal-based supper) in the total sample (*N* = 355).

Outcome variable: episodic recognition memory score										
	B	t	*p*	95% CI for B		SE B	β	F statistics	R^2^	ΔR^2^
				LL	UL					
Model 1										
**Step 1**			< 0.001					14.334	0.039	
Constant	3.465	30.125	< 0.001	3.239	3.692	0.115				
Age	−0.01	−3.786	< 0.001	−0.015	−0.005	0.003	−0.198			
**Step 2**			< 0.001					12.356	0.066	0.027
Constant	3.584	29.954	< 0.001	3.349	3.82	−0.12				
Age	−0.011	−4.265	< 0.001	−0.017	−0.006	0.003	−0.222			
Western-style supper	−0.219	−3.164	0.002	−0.354	−0.083	0.069	−0.165			
Model 2										
**Step 1**			< 0.001					14.334	0.039	
Constant	3.465	30.125	< 0.001	3.239	3.692	0.115				
Age	−0.01	−3.786	< 0.001	−0.015	−0.005	0.003	−0.198			
**Step 2**			< 0.001					11.389	0.061	0.022
Constant	3.563	29.964	< 0.001	3.329	3.797	0.119				
Age	−0.009	−3.354	< 0.001	−0.014	−0.004	0.003	−0.175			
Animal-based supper	−0.104	−2.856	0.005	−0.176	−0.032	0.036	−0.149			
Model 3										
**Step 1**			< 0.001					14.334	0.039	
Constant	3.465	30.125	< 0.001	3.239	3.692	0.115				
Age	−0.01	−3.786	< 0.001	−0.015	−0.005	0.003	−0.198			
**Step 2**			< 0.001					8.682	0.09	0.051
Constant	3.69	29.662	< 0.001	3.445	3.934	0.124				
Age	−0.01	−3.849	< 0.001	−0.016	−0.005	0.003	−0.201			
Western-style supper	−0.209	−2.187	0.029	−0.396	−0.021	0.095	−0.157			
Animal-based supper	−0.104	−2.549	0.011	−0.184	−0.024	0.041	−0.149			
Western-style supper × Animal-based supper	−0.02	−0.316	0.753	−0.143	0.103	0.063	−0.024			

B, unstandardized regression coefficient; SE B, standard error of the unstandardized regression coefficient; β = standardized regression coefficient; ΔR2, change in the proportion of variance explained relative to Step 1. The dependent variable was the d' score from the episodic recognition memory task.

We also found that the negative association between a Western-style supper and episodic recognition memory performance varies depending on age. While this relationship was significant overall (*B* = −0.14; *p* = 0.051), it became more pronounced in younger individuals. Specifically, for those aged 40 and older, the association remained significant but relatively weak (*B* = −0.140; *p* = 0.05). However, as age decreased, the negative impact of a Western-style supper on episodic recognition memory became stronger, reaching its most pronounced effect at age 20 (*B* = −0.391; *p* < 0.001). This suggests that younger individuals may be more vulnerable to the potential negative effects of a Western-style supper on episodic recognition memory.

In contrast, the negative association between an animal-based supper and episodic recognition memory performance remained consistent across different age groups. While individuals who consumed more animal-based foods at supper tended to have lower episodic recognition memory scores (*B* = −0.098; *p* = 0.007), this relationship did not significantly change with age (interaction effect animal-based supper × age; *B* = 0.002; *p* = 0.496). These findings highlight that while both dietary patterns may be linked to episodic recognition memory, age plays a role in shaping the effects of a Western-style supper but not an animal-based supper. Full regression coefficients and standard errors are presented in Tables 9, 10.

**Table 9:** Summary of linear regression models predicting episodic recognition performance from age, adherence to a western-style supper, and their interaction (Western-style supper × age) in the total sample (*N* = 355).

Outcome variable: episodic recognition memory score								
	B	SE	t	*p*	95% CI for B		F statistics	R^2^
					LL	UL		
**Model summary**				< 0.001			10.388	0.13
Constant	2.509	0.133	18.908	< 0.001	2.248	2.77		
Predictors								
Western-style supper	−0.14	0.071	−1.96	0.051	−0.28	0.001		
Age	−0.011	0.003	−3.919	< 0.001	−0.016	−0.005		
Western-style supper × Age	0.012	0.005	2.441	0.015	0.002	0.022		
Covariates								
Sex	0.451	0.123	3.663	< 0.001	0.209	0.693		
Education	0.226	0.097	2.334	0.02	0.036	0.417		

B, unstandardized regression coefficient; SE B, standard error of the unstandardized regression coefficient; β, standardized regression coefficient. The dependent variable was the d' score from the episodic recognition memory task.

**Table 10:** Summary of linear regression models predicting episodic recognition performance from age, adherence to animal-based supper, and their interaction (animal-based supper × age) in the total sample (*N* = 355).

Outcome variable: episodic recognition memory score								
	B	SE	t	*p*	95% CI for B		F statistics	R^2^
					LL	UL		
**Model summary**				< 0.001			8.91	0.113
Constant	2.475	0.135	18.312	< 0.001	2.209	2.741		
Predictors								
Animal-based supper	−0.098	0.036	−2.737	0.007	−0.168	−0.027		
Age	−0.01	0.003	−3.669	< 0.001	−0.015	−0.005		
Animal-based supper × age	0.002	0.002	0.681	0.496	−0.003	0.006		
Covariates								
Sex	0.435	0.124	3.513	< 0.001	0.191	0.678		
Education	0.26	0.099	2.626	0.009	0.065	0.455		

B, unstandardized regression coefficient; SE B, standard error of the unstandardized regression coefficient; β, standardized regression coefficient. The dependent variable was the d' score from the episodic recognition memory task.

### Milk-based products at supper are the strongest predictor of episodic recognition memory performance

Moving forward, we conducted a regression analysis to examine the composition of an animal-based supper in greater detail and assess the relative impact of its components on an objective measure of episodic recognition memory, while controlling for age. Five supper components were considered: (1) meat and cold cuts, (2) fish, (3) eggs, (4) milk-based products (e.g., milk, yogurt, kefir), and (5) dairy (e.g., cheese, cottage cheese). Among the five components, only milk-based products emerged as a significant predictor. The consumption of milk-based products at supper was associated with a further decline in memory performance (*B* = −0.216; *p* = 0.031), which explained 5.2% of the variance in episodic recognition memory. The prediction equation was: Episodic recognition memory score = 3.525 – 0.010 × age – 0.216 × milk-based products consumption. This suggests that individuals who consumed milk-based products at supper exhibited a reduction of approximately 0.22 points in episodic recognition memory performance compared to those who did not. Full regression coefficients and standard errors are presented in Table 11.

**Table 11:** Summary of linear stepwise regression analysis for individual components of animal-based supper predicting overall subjective memory index in the total sample (*N* = 355).

Stepwise regression on episodic recognition memory score										
	B	t	*p*	95% CI for B		SE B	β	F statistics	R^2^	ΔR^2^
				LL	UL					
**Step 1**			< 0.001					14.334	0.039	
Constant	3,465	30.125	< 0.001	3.239	3.692	0.115				
Age	−0,01	−3.786	< 0.001	−0.015	−0.005	0.003	−0.198			
**Step 2**			< 0.001					9.589	0.052	0.013
Constant	3,525	29.953	< 0.001	3.293	3.756	0.118				
Age	−0.01	−3.902	< 0.001	−0.016	−0.005	0.003	−0.203			
Milk-based products	−0.216	−2.166	0.031	−0.413	−0.02	0.1	−0.113			
Excluded variables										
Eggs		−0.541	0.589				−0.028			
Meat (processed/cold cuts)		−1.213	0.226				−0.063			
Dairy (cheese)		−1.441	0.15				−0.076			
Fish		−1.966	0.05				−0.104			

B, unstandardized regression coefficient; SE B, standard error of the unstandardized regression coefficient; β, standardized regression coefficient; ΔR2, change in the proportion of variance explained relative to Step 1. The dependent variable was the d' score from the episodic recognition memory task.

### Sweets at supper are the strongest predictor of the episodic recognition memory performance

Moving forward, we conducted a regression analysis to examine the composition of a Western-style supper in greater detail and assess the relative impact of its components on an objective measure of episodic recognition memory, while controlling for age. Three supper components were considered: (1) sweets and desserts, (2) salty snacks, and (3) alcohol. Among these, only sweets and desserts emerged as a significant predictor. The consumption of sweets and desserts was associated with a further decline in memory performance (*B* = −0.403; *p* < 0.001), which explained 6.9% of the variance in episodic recognition memory. The prediction equation was: Episodic recognition memory score = 3.539 – 0.011 × age – 0.403 × sweets and desserts consumption. This suggests that individuals who consumed sweets and desserts at supper exhibited a reduction of approximately 0.40 points in episodic recognition memory performance compared to those who did not. Full regression coefficients and standard errors are presented in Table 12.

**Table 12:** Summary of linear stepwise regression analysis for individual components of western-style supper predicting overall subjective memory index in the total sample (*N* = 355).

Stepwise regression on episodic recognition memory score										
	B	t	*p*	95% CI for B		SE B	β	F statistics	R^2^	ΔR^2^
				LL	UL					
**Step 1**			< 0.001					14.334	0.039	
Constant	3.465	30.125	< 0.001	3.239	3.692	0.115				
Age	−0.01	−3.786	< 0.001	−0.015	−0.005	0.003	−0.198			
**Step 2**			< 0.001					13.131	0.069	0.03
Constant	3.539	30.66	< 0.001	3.312	3.766	0.115				
Age	−0.011	−4	< 0.001	−0.016	−0.005	0.003	−0.206			
Sweets	−0.403	−3.391	< 0.001	−0.637	−0.169	0.119	−0.175			
Excluded variables										
Alcohol		−0.205	0.838				−0.011			
Salty snacks		−1.018	0.309				−0.055			

B, unstandardized regression coefficient; SE B, standard error of the unstandardized regression coefficient; β = standardized regression coefficient; ΔR2, change in the proportion of variance explained relative to Step 1. The dependent variable was the d' score from the episodic recognition memory task.

Finally, to gain a more comprehensive understanding of whether consuming foods with potential adverse health effects at supper is negatively associated with memory - regardless of participants' adherence to a plant-based diet - we examined the relationships between Western-style and animal-based suppers and episodic recognition memory scores. We also included interaction terms for Western-style supper × plant-based diet and animal-based supper × plant-based diet to assess whether these supper types interact while controlling for age. Neither the interaction between a plant-based diet and a Western-style supper (interaction effect Western-style supper × plant-based diet; *B* = 0.04; *p* = 0.587) nor the interaction between a plant-based diet and an animal-based supper (interaction effect animal-based supper × plant-based diet; *B* = 0.022; *p* = 0.556) was significant. This indicates that a plant-based diet does not mitigate the negative associations between unhealthy supper choices and memory performance. While a plant-based diet is often linked to cognitive benefits, it does not counteract the detrimental effects of consuming unhealthy foods at supper. Full regression coefficients and standard errors are presented in Table 13.

**Table 13:** Summary of linear regression models predicting episodic recognition performance from age, adherence to plant-based diet, western-style and animal-based supper and their interactions (western-style supper × age and animal-based supper × age) in the total sample (*N* = 355).

Western-style supper										
Outcome variable: episodic recognition memory score										
	B	t	*p*	95% CI for B		SE B	β	F statistics	R^2^	ΔR^2^
				LL	UL					
**Step 1**			< 0.001					14.334	0.039	0.039
Constant	3.465	30.125	< 0.001	3.239	3.692	0.115				
Age	−0.01	−3.786	< 0.001	−0.015	−0.005	0.003	−0.198			
**Step 2**			< 0.001					6.442	0.069	0.03
Constant	3.605	29.49	< 0.001	3.364	3.845	0.122				
Age	−0.012	−4.337	< 0.001	−0.017	−0.006	0.003	−0.232			
Western-style supper	−0.213	−3.055	0.002	−0.35	−0.76	0.07	−0.16			
Plant-based diet	0.027	0.576	0.565	−0.66	0.121	0.047	0.034			
Western-style supper × Plant-based diet	0.04	0.544	0.587	−0.106	0.187	0.074	0.032			
Animal-based supper										
Outcome variable: episodic recognition memory score										
	B	t	*p*	95% CI for B		SE B	β	F statistics	R^2^	ΔR^2^
				LL	UL					
**Step 1**			< 0.001					14.334	0.039	0.039
Constant	3.465	30.125	< 0.001	3.239	3.692	0.115				
Age	−0.01	−3.786	< 0.001	−0.015	−0.005	0.003	−0.198			
**Step 2**			< 0.001					5.998	0.064	0.025
Constant	3.585	29.487	< 0.001	3.346	3.824	0.122				
Age	−0.009	−3.464	< 0.001	−0.015	−0.004	0.003	−0.186			
Animal-based supper	−0.104	−2.861	0.004	−0.176	−0.033	0.036	−0.15			
Plant-based diet	0.013	0.197	0.844	−0.114	0.139	0.064	0.016			
Animal-based supper × Plant-based diet	0.022	0.59	0.556	−0.052	0.097	0.038	0.046			

B, unstandardized regression coefficient; SE B, standard error of the unstandardized regression coefficient; β, standardized regression coefficient. The dependent variable was the d' score from the episodic recognition memory task.

### Neither animal-based supper nor western-style supper is associated with short-term memory performance

To explore whether Western-style and animal-based supper are associated with short-term memory, we examined their relationship with short-term memory capacity scores. We also considered whether these two supper types interact with each other and accounted for age as a control factor. Adding Western-style and animal-based suppers to the model, did not meaningfully improve the explanation of short-term memory. Neither Western-style supper (*B* = 0.347; *p* = 0.169) nor animal-based supper (*B* = −0.062; *p* = 0.565) alone or in combination (interaction effect Western-style supper × animal-based supper; *B* = −0.204; *p* = 0.218), showed a clear link to short-term memory capacity scores, suggesting that these supper types do not independently affect short-term memory performance. Full regression coefficients and standard errors are presented in Table 14.

**Table 14:** Summary of linear regression models predicting short-term memory performance from age, adherence to a western-style and animal-based supper and their interaction (western-style supper × animal-based supper) in the total sample (*N* = 355).

Outcome variable: short-term memory capacity										
	B	t	*p*	95% CI for B		SE B	β	F statistics	R^2^	ΔR^2^
				LL	UL					
**Step 1**			< 0.001					41.676	0.106	0.106
Constant	6.112	20.581	< 0.001	5.528	6.696	0,.97				
Age	−0.044	−6.456	< 0.001	−0.058	−0.031	0.007	−0.325			
**Step 2**			< 0.001					11.417	0.115	0.01
Constant	6.116	18.63	< 0.001	5.47	6.761	0.328				
Age	−0.043	−6.116	< 0.001	−0.057	−0.029	0.007	−0.316			
Western-style supper	0.347	1.378	0.169	−0.148	0.842	0.252	0.098			
Animal-based supper	−0.062	−0.576	0.565	−0.274	0.15	0.108	−0.033			
Western-style supper × Animal-based supper	−0.204	−1.234	0.218	−0.528	0.121	0.165	−0.092			

B, unstandardized regression coefficient; SE B, standard error of the unstandardized regression coefficient; β, standardized regression coefficient; ΔR2, change in the proportion of variance explained relative to Step 1. The dependent variable was the capacity score from short-term memory task.

In Study 1, subjective memory ratings were lower among individuals who consumed more foods with potential adverse health effects throughout the day. This effect was especially pronounced at supper where each additional food item with unhealthy potential led to nearly a five-point drop in self-assessed memory scores. Among these, frequent cheese consumption emerged as the strongest predictor of poorer memory ratings with a reduction of about thirteen points. While in Study 2 objective memory assessments revealed that although adherence to a Western-style dietary pattern did not significantly correlate with episodic recognition memory performance, it was linked to lower short-term memory capacity especially among older participants. Additionally, an animal-based diet showed no clear relation to episodic recognition memory yet its negative association with short-term memory became evident from middle age onward. Further analyses demonstrated that both Western-style and animal-based suppers independently contributed to lower episodic recognition memory performance with the detrimental effect of a Western-style supper being more pronounced in younger individuals. Within these supper patterns, the consumption of sweets and desserts as well as milk-based products were identified as the most damaging components. Interestingly, even though an adherence to a plant-based diet is generally associated with cognitive benefits, it did not counteract the negative effects of unhealthy supper choices on memory performance. These findings collectively illustrate a complex picture in which dietary patterns and specific meal-based components are associated with both subjective and objective memory outcomes across the lifespan.

## Discussion

### Link between diet and memory

**Figure 5:**
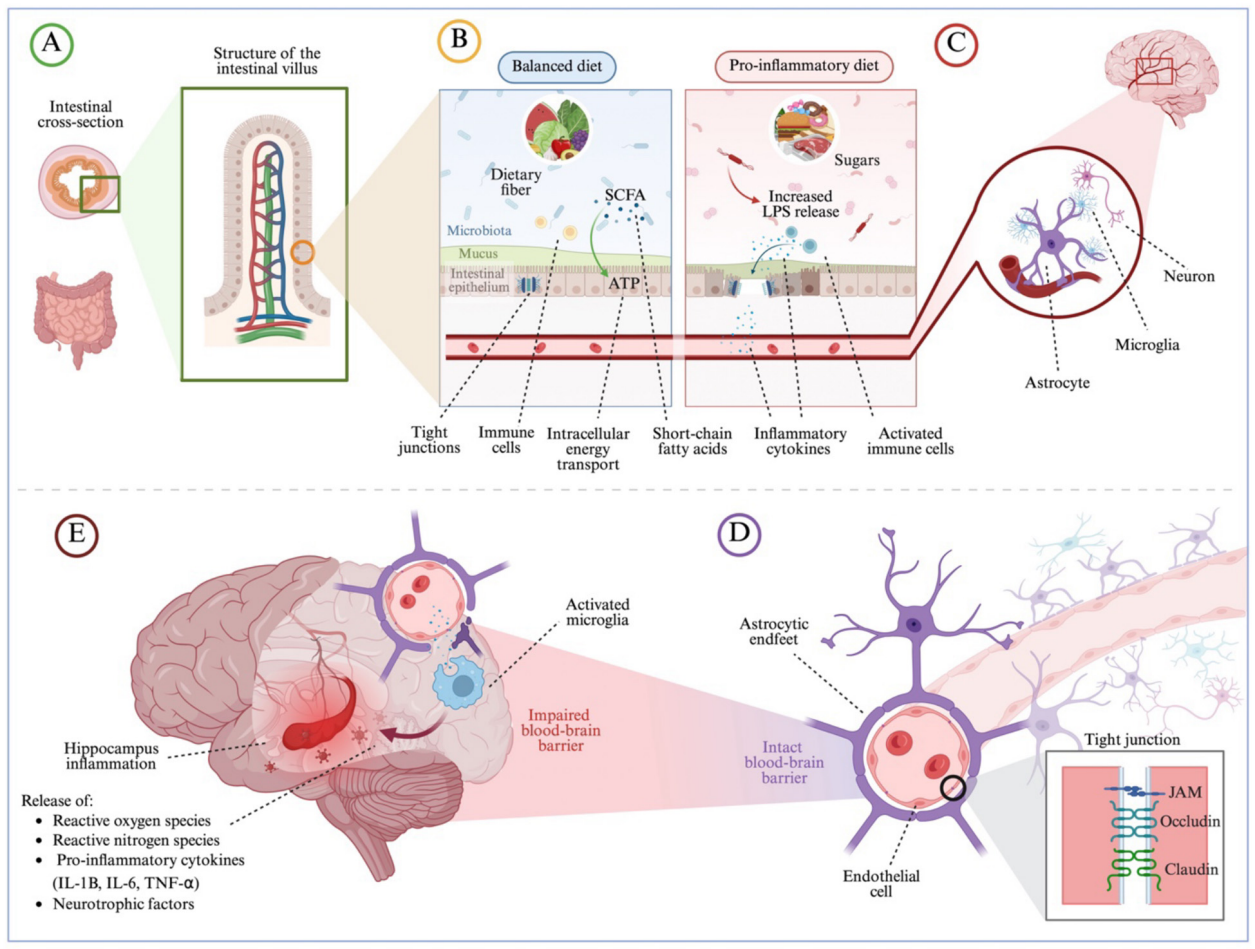
Diet induced gut dysbiosis triggers neuroinflammation and hippocampal dysfunction via the gut-brain axis. Hippocampal inflammation arises from diet-induced gut dysbiosis, which reduces short-chain fatty acid (SCFA) production and promotes the overgrowth of Gram-negative bacteria. Upon bacterial death, lipopolysaccharides (LPS) are released, triggering immune cells in the gut to produce pro-inflammatory cytokines (37). These cytokines circulate systemically, compromising the blood-brain barrier (BBB) by damaging endothelial cells and disrupting tight junctions. Once the BBB becomes permeable, LPS and inflammatory molecules infiltrate the brain, activating microglia. The resulting microglial response—characterized by oxidative stress and further cytokine release—sustains neuroinflammation, impairing synaptic plasticity, neurogenesis, and cognitive functions such as learning and memory. Panel Descriptions: **(A)** The anatomical structure of the intestinal lining, highlighting villi and crypts where nutrient absorption and microbial interactions occur. **(B)** A healthy gut environment featuring dietary fiber, diverse microbiota, an intact mucus layer, and functional intestinal epithelium, all supporting SCFA production. **(C)** The effects of a Western diet: reduced SCFAs, increased sugar metabolism, and overgrowth of Gram-negative bacteria leading to LPS release and immune activation. **(D)** The intact BBB, maintaining brain homeostasis. **(E)** Consequences of BBB disruption on hippocampal neurons, resulting in inflammation and impaired neuronal function. Created with BioRender.com.

Dietary patterns significantly influence both the composition and regulation of the gut microbiome, the community of microorganisms living in the gastrointestinal tract. Nearly every ingested substance is metabolized to some degree by these microbes. This dynamic triangular interaction among diet, microbiota composition, and cognitive function may help explain the range of beneficial and adverse effects that diet has on brain health (9). Importantly, although performance on memory tasks cannot be taken as direct evidence of hippocampal involvement and is more appropriately understood as reflecting processes typically supported by medial temporal lobe systems, the pathway described here offers a plausible mechanistic account because the hippocampus is one of the structures within these systems that may be particularly sensitive to diet-induced inflammation, blood–brain barrier disruption, and metabolic imbalance. To illustrate this proposed pathway (Figure 5.), the WD disrupts gut microbiota, reducing the production of short-chain fatty acids (SCFAs), which help maintain gut integrity (B). As SCFA levels decline, Gram-negative bacteria proliferate. Upon their death, lipopolysaccharides (LPS) are released through lysis. LPS activates immune cells in the gut, prompting the release of pro-inflammatory cytokines (37). These cytokines travel through the bloodstream, weakening the blood-brain barrier (BBB) by impairing endothelial cells and disrupting tight junctions, ultimately increasing BBB permeability (E). The hippocampal BBB has recently been suggested to be weaker than those in other brain regions, making it more vulnerable to disruptions in the mechanisms that maintain its internal environment (11). As a result, LPS and inflammatory mediators infiltrate the brain, triggering the activation of microglia, the brain's resident immune cells. Activated microglia release additional pro-inflammatory cytokines, further exacerbating neuroinflammation. This persistent inflammatory state in the hippocampus disrupts synaptic plasticity, impairs neurogenesis, and compromises neuronal function, ultimately leading to deficits in learning, memory, and cognitive flexibility. Through this cascade of events, the Western diet promotes hippocampal dysfunction, linking poor dietary habits to episodic recognition memory decline and cognitive impairment.

An additional possibility is that the observed association may partly reflect sleep-related pathways. Although sleep was not measured in the present study, poorer overall diet quality, particularly dietary patterns higher in saturated fat and refined or free sugars and lower in fiber, has been associated with poorer sleep quality (38, 39). Poorer sleep, in turn, has been linked to worse memory functioning, including episodic memory performance (40, 41). From this perspective, unhealthy supper choices may contribute to poorer memory performance at least partly by creating conditions less favorable for sleep.

### Supper choices and episodic recognition memory: most pronounced associations in young adults

Our results are consistent with previous findings suggesting that Western-style dietary patterns may be associated with poorer memory performance, particularly on tasks supported by medial temporal lobe systems. We found that unhealthy supper choices were associated with poorer episodic recognition memory, while short-term memory remained unaffected. Although this negative association was evident across all age groups, it was most pronounced among individuals around the age of 20, suggesting that younger individuals might be particularly vulnerable to these adverse dietary effects. These results align with investigations in rodent models (42), which have demonstrated that early-life exposure to high-fat diets (accounting for 40–65% of caloric intake) or diets rich in simple sugars (such as sucrose or high-fructose corn syrup) can impair hippocampus-dependent learning and memory processes. In some cases, these deficits have persisted into adulthood despite later dietary interventions (42). Another study also showed that the beneficial effect of the Mediterranean Diet on cognitive decline was observed only in individuals with low adherence to a WD, and not in those with high adherence (43). Similarly, our study found that even adherence to a plant-based diet did not counteract the negative impact of unhealthy supper choices on memory performance.

### Western diet correlates with various cognitive domains

Quite unexpectedly, our findings revealed that an overall WD did not show a negative association with episodic recognition memory scores. Instead, it showed a negative correlation with short-term memory scores. It should be emphasized that many studies have focused on the detrimental effects of the WD diet on memory performance, particularly on tasks engaging medial temporal lobe systems (13–21), a close review of the literature indicates declines across various cognitive domains (44–46), including executive function and working-memory functioning (16). Thus, although the hippocampus may be particularly vulnerable, other brain regions might also be at risk. Moreover, individuals may perceive that their memory is not in optimal condition. We found that the lower participants rated their own memory function, the higher their scores were on the unhealthy dietary index. Similar results were reported in another study, in which self-reported everyday memory failures were associated with habitual dietary intake of fat and sugar (14).

### Animal-based diet and memory: age matters

It is noteworthy that the WD primarily consists of animal products, such as red meat and dairy, which are major sources of saturated fat (47). Our findings revealed a negative correlation between higher adherence to an animal-based diet and short-term memory performance, with this association becoming more pronounced from age 45 onward. This observation is supported by previous research showing that high red meat intake is associated with an increased risk of cognitive impairment (48). Moreover, the consumption of full-fat dairy products, red meat, and processed meat has been linked to poorer executive function and overall cognitive performance (49). In contrast, a lower intake of dairy products, meat, and saturated fatty acids appears to reduce the risk of developing mild cognitive impairment (MCI) (Yuan et al., 2016) and the likelihood of progression from MCI to Alzheimer's disease (AD) (50). However, our results should be interpreted with caution, as the animal-based diet in our study includes both red and white meat, both high-quality and ultra-processed, as well as dairy from cottage cheese to processed cheese spreads. Notably, a recent study showed that the type of meat matters: red meat and animal fat consumption were negatively correlated with cognitive performance, whereas white meat and fish consumption were positively related to memory (51). Our results suggest that higher consumption of animal-based products, regardless of quality or type, is associated with poorer short-term memory performance in adults aged 45 years and older.

### Fermented dairy and its ambiguous cognitive impact

In our study, milk-based products were clustered as milk and milk-based drinks (e.g., yogurt, kefir), which include both fresh milk and fermented dairy items. Fermented dairy products have attracted recent attention, foods made with beneficial bacteria (52) that can survive gastrointestinal transit, thereby enhancing gut microbial diversity and increasing the abundance of *Lactobacillus*. This bacterial boost may lead to neuromodulatory effects on cognition via the production of short-chain fatty acids (SCFAs), which play a crucial role in maintaining both the blood–brain barrier and intestinal barrier. Intervention studies on fermented dairy have generally reported improvements in cognitive functioning, although the evidence is not entirely consistent. Several investigations [e.g., (53–57)) have observed enhanced cognitive outcomes relative to placebo, whereas other studies noted no significant differences in MMSE scores (58). Observational research further complicates the picture. For example, while one study found that fermented dairy consumption was linked to better executive functioning (59), another reported that higher intake of fermented dairy products correlated with lower MMSE scores (60). Similarly, studies on dairy consumption in general yield mixed results. Some research indicates that higher consumption of skimmed dairy or buttermilk, as well as daily dairy intake, is associated with superior cognitive performance (59, 61). Conversely, other studies have documented that greater intake of total milk or whole-fat milk is related to accelerated declines in global cognitive function (62), faster cognitive decline from midlife to late-life (63), and poorer verbal memory performance (64). Our findings resonate with these latter observations, suggesting a negative correlation between cognitive performance and the consumption of milk-based drinks, including those that are fermented. A few methodological limitations in our study may have contributed to these results. Firstly, we did not differentiate between milk and fermented milk, despite the possibility that these products might exert distinct effects on cognition. Secondly, we did not control the quantity of dairy consumed at supper. This is critical because meta-analyses suggest that moderate dairy intake may lower the risk of cognitive decline or dementia (65), yet the optimal consumption levels appear to differ by region. For instance, studies conducted in Asia have reported beneficial effects at relatively low levels of dairy intake (29–165 g/day) (66–70), whereas European studies, where dairy consumption is significantly higher (170–711 g/day), generally do not find such protective associations (64, 71–76). It is plausible that our participants are consuming dairy in amounts that exceed the threshold for cognitive benefit. European dietary guidelines typically recommend 2–4 servings daily (approximately 250–500 ml or 300–450 g, up to 1/2 liter per day) (77), which is considerably higher than the amounts associated with beneficial effects in Asian studies. Future research should investigate different quantities of dairy consumption and their effects on cognition. Lastly, we did not account for whether participants consumed these dairy products with added sugar. A systematic review and meta-analysis showed that both cohort and cross-sectional studies found significant positive correlations between added sugar consumption and the risk of cognitive impairment (78). Additionally, consumption of sugary dairy was associated with a 4.4% increase in the risk of cognitive decline. Moreover, replacing vegetables with sugary dairy products increased the risk of cognitive decline by 6.9% (79). We hypothesize that the presence of added sugars in flavored milk drinks, yogurts, or kefir might attenuate the potential benefits derived from the live bacteria. Future research should aim to disentangle the effects of dairy type and sugar content to better understand their collective impact on cognitive function.

### Further limitations to consider

Several additional methodological limitations should be acknowledged when interpreting our findings. First, our study relied on self-reported dietary data, which are subject to recall bias and social desirability effects. Participants may have under- or over-reported their consumption of certain food items, particularly those perceived as unhealthy.

Second, the study was conducted entirely online, which limited our ability to verify participants' responses and control the testing environment. Online assessment is inherently less standardized and may therefore introduce additional variability. Nevertheless, several procedures were implemented to enhance data quality despite these constraints. These included embedded survey control questions, such as asking participants to identify a topic not yet covered in the questionnaire, as well as instructed-response items within the FFQ. In addition, during the learning phase of the Old/New Recognition task, participants completed eight interim attention-check questions concerning the previously presented stimulus. Participants who failed these control procedures or scored below the predefined threshold were excluded from further analyses.

Third, the cross-sectional design of our study limits causal inference. Although we observed associations between diet and memory, the direction of these relationships cannot be established. Moreover, Study 2 was based on a predominantly female sample (311 women, 44 men), which may reduce the generalizability of the findings, particularly to men. Although sex was included as a covariate in the regression models, the marked imbalance between women and men precluded robust sex-stratified analyses and prevents strong conclusions regarding potential sex-specific effects.

### The importance of meal-specific dietary analysis

These findings carry important implications and signal a necessary shift in how dietary research is approached. Our study highlights the significance of examining individual meals, especially supper, in isolation, rather than relying solely on broad, overall dietary patterns. By focusing on meal-specific components, we uncover nuanced associations with memory performance that might otherwise be obscured in aggregate analyses. This meal-based approach challenges conventional paradigms in nutritional neuroscience and calls for a more granular, time-sensitive examination of food intake. It not only refines our understanding of how specific food choices at mealtimes influence cognition, but also lays the groundwork for future research and targeted dietary interventions aimed at promoting brain health across the lifespan.

## Conclusion

In conclusion, the present findings indicate that the relationship between diet and memory may depend not only on overall dietary patterns, but also on the specific timing and composition of meals. Across two independent studies, supper emerged as the meal most consistently associated with memory-related outcomes, linking higher consumption of foods with potential adverse health effects to both poorer self-perceived memory functioning in everyday life and lower episodic recognition memory performance. These associations were further refined at the food-component level, with higher consumption of cheese at supper predicting poorer self-rated memory, while more frequent consumption of milk-based products, as well as sweets and desserts, at supper emerged as the strongest predictors of poorer objective episodic memory performance. The observation that the negative association between Western-style supper and episodic recognition memory was more pronounced in younger adults additionally suggests that the cognitive correlates of evening eating habits may not be uniform across the lifespan. Together, these results underscore the value of a meal-specific approach in nutritional cognitive research. Considering not only what people eat, but also when they eat it, may offer a more nuanced understanding of diet–memory associations and help identify more precise targets for future dietary prevention and intervention strategies.

## Data Availability

The datasets presented in this study can be found in online repositories. The names of the repository/repositories and accession number(s) can be found below: https://share.swps.edu.pl/entities/dataset/e4d58994-42f1-45db-9ed9-0f19b550d504.
