# Supplementary material for: Meal-specific dietary patterns relate to memory functioning
Source: Front Nutr. 2026 Apr 28;13:1760033. doi: 10.3389/fnut.2026.1760033 (PMC13160797; doi:10.3389/fnut.2026.1760033)
Supplement: Supplementary file 1 [file Table_1.docx]

**Supplementary materials:
Table 1S**. PCA analysis of FFQ data. Bold values indicate factor loading of greater than 0.4. Excluded variables: Oil, Tea, Coffee, Mayonnaise, Sweet fruit preserves, Margarine, Wine, Alcohol, Beer

| **FFQ components** | **Animal-based diet** | **Plant-based diet** | **Western-style diet** |
| --- | --- | --- | --- |
| Poultry meat (e.g., hen, chicken, duck, turkey meat) | **0.747** | -0.111 | 0.023 |
| Red meat (e.g., pork, beef, veal) | **0.734** | -0.197 | 0.034 |
| Mixed meat dishes (e.g., mixed meat dishes with additives, such as hunter’s stew (in polish: bigos), stuffed cabbage rolls) | **0.704** | -0.08 | 0.172 |
| Sausages (e.g., kabanos sausages, luncheon meat, frankfurters) | **0.7** | -0.134 | 0.176 |
| High-quality cold cuts (e.g., high-quality poultry and pork-beef cold cuts) | **0.693** | -0.118 | -0.019 |
| Offal and processed meat products (e.g., liver, blood sausage, head cheese, pâtés, bacon) | **0.666** | -0.088 | -0.025 |
| Other animal fats (e.g., lard, fat from cooked dishes) | **0.547** | -0.107 | 0.041 |
| Eggs (e.g., hard-boiled, soft-boiled, scrambled eggs) | **0.536** | 0.17 | -0.057 |
| Fish and seafood (e.g., smoked, marinated, in oil, in cream sauce, canned, fried, boiled fish) | **0.515** | 0.168 | -0.21 |
| Butter | **0.514** | 0.021 | 0.065 |
| Milk and dairy drinks (e.g., milk, milk-based soups, milk drinks, yogurt, kefir, buttermilk) | **0.502** | 0.16 | 0.113 |
| Cream (e.g., various creams) | **0.467** | 0.105 | 0.266 |
| Cottage cheese (e.g., various cottage cheeses, flavoured and plain) | **0.45** | 0.389 | 0.027 |
| Yellow-orange vegetables (e.g., carrot, pepper) | -0.045 | **0.778** | -0.04 |
| Other vegetables (e.g., celery, corn, radish, salads, mixed vegetable salads) | -0.062 | **0.662** | -0.039 |
| Leafy green vegetables (e.g. various lettuces, leek) | -0.108 | **0.655** | -0.123 |
| Cruciferous vegetables (e.g. broccoli, brussels sprouts, fresh cabbage and sauerkraut, cauliflower) | -0.016 | **0.631** | -0.13 |
| Berry fruits (e.g. strawberries, whortleberries, raspberries, blueberries) | 0.134 | **0.592** | -0.143 |
| Fruits (e.g. apples, pears, plums, cherries, bananas, oranges, grapefruit, kiwi, peaches, grapes) | 0.038 | **0.586** | 0.03 |
| Leguminous vegetables (e.g. string beans, green peas, beans, peas, lentils, broad beans) | -0.358 | **0.573** | 0,011 |
| Tomatoes (e.g. tomatoes, tomato juice) | 0.048 | **0.533** | 0.033 |
| Soups (e.g. vegetable soups, broths) | 0.088 | **0.485** | 0.046 |
| Herbal and fruit tea | -0.044 | **0.469** | -0.056 |
| Nuts (e.g. various nuts, peanut butter, chocolate and nut cream) | -0.012 | **0.456** | -0.089 |
| Whole-grain products (e.g. wholemeal wheat bread, rye bread, bread with grains, pumpernickel, grahams, buckwheat gains) | -0.076 | **0.43** | 0.139 |
| Fruit and vegetable juices (e.g. carrot juice) | 0.039 | **0.42** | 0.157 |
| French fries, potato pancakes (e.g., French fries, potato pancakes, potato babka) | 0.049 | -0.036 | **0.686** |
| Flour-based dishes (e.g., pizza, dumplings, pancakes, lazy dumplings (in polish: kluski leniwe), Silesian dumplings (in polish: kluski śląskie)) | 0.037 | 0.039 | **0.684** |
| Salty snacks (e.g., sticks, crisps, roasted corn, chips) | -0.092 | -0.201 | **0.66** |
| Sugar-sweetened carbonated soft drinks (e.g., carbonated drinks such as Coca-cola, Fanta, Sprite, carbonated fruit drinks) | -0.062 | -0.14 | **0.653** |
| Highly refined grain products (e.g., white wheat bread, rye bread, wheat-rye bread, toast bread, bread rolls, butter rolls and croissants, French croissants, sweet rolls, pasta, rice, highly refined grains) | 0.092 | -0.038 | **0.644** |
| Sweets and desserts (e.g., sugar, honey, biscuits, cream-filled cookies, shortcrust pastries, semi-shortcrust pastries, fruit-filled pastries, yeast buns, cheesecake, doughnuts, poppy seed roll, various chocolates, ice cream, pudding) | 0.042 | -0.078 | **0.613** |
| Sauces (e.g., for meat, potatoes, grains, dishes) | 0.189 | -0.048 | **0.519** |
| Energy drinks (e.g., Red-Bull, Tiger, Burn) | -0.108 | -0.211 | **0.507** |
| Ready-to-eat breakfast cereals (e.g., uncooked milk additives: muesli, cornflakes) | 0.038 | 0.078 | **0.491** |
| Potatoes (e.g., potatoes, potato dumplings (in polish: kopytka), dumplings (in polish: pyzy) | 0.121 | 0.248 | **0.482** |
| Fruit juices (e.g., apple, orange, grapefruit, currant, other juices, multifruit) | 0.044 | 0.133 | **0.469** |
| Cheeses (e.g., rennet cheese, cheeses spread) | 0.309 | 0.077 | **0.399** |
